# Thermostable recombinant xylanases from *Nonomuraea flexuosa *and *Thermoascus aurantiacus *show distinct properties in the hydrolysis of xylans and pretreated wheat straw

**DOI:** 10.1186/1754-6834-4-12

**Published:** 2011-05-18

**Authors:** Junhua Zhang, Matti Siika-aho, Terhi Puranen, Ming Tang, Maija Tenkanen, Liisa Viikari

**Affiliations:** 1 College of Forestry, Northwest A&F University, 3 Taicheng Road, Yangling 712100, China; 2 VTT Technical Research Centre of Finland, P.O. Box 1000, FIN-02044 Espoo, Finland; 3 Roal Oy, Tykkimäentie 15, FIN-05200, Rajamäki, Finland; 4 Department of Food and Environmental Sciences, University of Helsinki, P.O. Box 27, FIN-00014 Helsinki, Finland

## Abstract

**Background:**

In the hydrolysis of lignocellulosic materials, thermostable enzymes decrease the amount of enzyme needed due to higher specific activity and elongate the hydrolysis time due to improved stability. For cost-efficient use of enzymes in large-scale industrial applications, high-level expression of enzymes in recombinant hosts is usually a prerequisite. The main aim of the present study was to compare the biochemical and hydrolytic properties of two thermostable recombinant glycosyl hydrolase families 10 and 11 (GH10 and GH11, respectively) xylanases with respect to their potential application in the hydrolysis of lignocellulosic substrates.

**Results:**

The xylanases from *Nonomuraea flexuosa *(Nf Xyn11A) and from *Thermoascus aurantiacus *(Ta Xyn10A) were purified by heat treatment and gel permeation chromatography. Ta Xyn10A exhibited higher hydrolytic efficiency than Nf Xyn11A toward birchwood glucuronoxylan, insoluble oat spelt arabinoxylan and hydrothermally pretreated wheat straw, and it produced more reducing sugars. Oligosaccharides from xylobiose to xylopentaose as well as higher degree of polymerization (DP) xylooligosaccharides (XOSs), but not xylose, were released during the initial hydrolysis of xylans by Nf Xyn11A, indicating its potential for the production of XOS. The mode of action of Nf Xyn11A and Ta Xyn10A on glucuronoxylan and arabinoxylan showed typical production patterns of endoxylanases belonging to GH11 and GH10, respectively.

**Conclusions:**

Because of its high catalytic activity and good thermostability, *T. aurantiacus *xylanase shows great potential for applications aimed at total hydrolysis of lignocellulosic materials for platform sugars, whereas *N. flexuosa *xylanase shows more significant potential for the production of XOSs.

## Background

Xylans represent a major source of plant cell wall polysaccharides with structural diversity and increasing application opportunities. Xylans in hardwoods and annual plants consist of a linear backbone of β-(1→4)-D-xylopyranosyl residues, substituted by α-L-arabinofuranosyl units in the positions of 2-*O*- and/or 3-*O*-, by 4-*O*-methyl-glucopyranosyl uronic acid in the position of 2-*O*, and/or by acetyl groups in the positions of 2-*O *and/or 3-*O *[1]. Furthermore, some of the arabinofuranosyl units may be esterified with ferulic or *p*-coumaric acids [2]. Complete hydrolysis of xylan involves the synergistic action of several main chain- and side group-cleaving enzymes, including endo-β-1,4-xylanases (EC 3.2.1.8), β-D-xylosidases (EC 3.2.1.37), α-L-arabinofuranosidases (EC 3.2.1.55), α-glucuronidases (EC 3.2.1.139), acetyl xylan esterases (EC 3.1.1.72) and feruloyl esterases (EC 3.1.1.73).

Endo-β-1,4-xylanases cleave randomly the internal β-1,4-glycosyl bonds in the xylan main chain. On the basis of the similarities in their amino acid sequences and hydrophobic cluster analysis, xylanases are currently classified into families 5, 8, 10, 11 and 43 of glycoside hydrolases [3]. Xylanases from family 10 (GH10) and family 11 (GH11) of glycoside hydrolases are the major and best-studied xylanases. Enzymes from these two families differ in their physicochemical properties, as well as in their action on polysaccharides and xylooligosaccharides (XOSs) [4,5]. GH10 xylanases have generally a higher molecular mass and lower isoelectric point than GH11 xylanases. GH 10 xylanases all have a catalytic domain which exhibits (β/α)_8 _architecture, whereas the GH11 xylanases have a β-jelly roll structure [6]. GH10 xylanases are capable of cleaving glycosidic linkages in the xylan main chain closer to the substituents, such as the 4-*O*-methyl-glucuronic acid, α-L-arabinofuranose and acetyl groups. As a result, shorter hydrolysis products are released from xylan by GH10 xylanases as reported by Biely *et al*. [4].

Xylanases have raised interest because of their potential applications in various industrial fields, including the pulp and paper industries, bioethanol production and the feed industry [6-9]. Xylanases have been found to be important in the total hydrolysis of hemicelluloses containing lignocellulosic materials. In bioethanol production processes from lignocellulosic materials, xylanases can improve the hydrolysis of cellulose into fermentable sugars by removing xylans restricting the access of cellulases to cellulose surfaces [10]. Thus, hydrolysis of pretreated lignocellulosic raw materials with even low residual xylan content by xylanases has been shown to improve the hydrolysis of cellulose significantly [10]. Whereas extensive research on the partial hydrolysis of xylans in cellulosic pulps has been carried out, only a few reports are available on the mechanisms and efficiencies of various xylanases on complex lignocellulosic substrates. Xylanases can also be used for the production of XOSs, which exhibit a wide range of biological activities, including prebiotic effects, antioxidative and antimicrobial activities, as well as plant growth regulatory effects [11,12]. Thus, XOSs show remarkable potential for practical utilization in many fields, including functional food, pharmaceuticals, feed formulations and agricultural applications. Enzymes with optimal catalytic properties for each application may be found among different families.

Thermostable enzymes have several generic advantages, allowing a decreased amount of enzyme needed because of higher specific activity and elongated hydrolysis time due to higher stability [9]. In addition, thermostable enzymes are generally more tolerant and allow more flexibility in process configurations. Thermostable xylanases can be obtained from many bacterial and fungal sources [6,8]. Several xylanases isolated from the thermophilic fungus *Thermoascus aurantiacus *belong to the GH10 family and have high specific activity and thermal stability. Xylanases from *T. aurantiacus *C-436 have been shown to be stable at 50°C for 12 weeks and to retain 90% of the initial activity. In addition, the half-lives of the xylanases at 70°C and 60°C were 1.5 hours and 4 days, respectively [13]. Two xylanases from *T. aurantiacus *IMI 216529 were reported to have half-lives of 88 and 41 minutes at 80°C [14]. Two family 11 xylanases purified from the thermophilic actinomycete *Nonomuraea flexuosa *(previously *Microtetraspora flexuosa*) SIIX had half-lives of about 5 minutes at 80°C in the absence of substrate and half-lives of about 25 minutes at 80°C in the presence of 1% substrate [15]. The xylanase genes *xyn11A *from *N. flexuosa *and *xyn10A *from *T. aurantiacus *have been cloned and expressed in *Trichoderma reesei *[16,17]. The three recombinant proteins from *N. flexuosa *(r33.4, r23.8 and r22.0 kDa) obtained were similar to each other in terms of temperature dependence, but the shorter polypeptides were more thermostable at 80°C than the full-length enzyme, especially at pH 5 [16]. Both of the two shorter polypeptides from *N. flexuosa *and the xylanase 10A from *T. aurantiacus *lacked the C-terminal cellulose binding module (CBM). There are no reports, however, on the hydrolytic properties of the thermostable recombinant xylanases from *N. flexuosa *or on those of the thermostable recombinant Xyn10A xylanase from *T. aurantiacus *expressed in *T. reesei*.

For cost-efficient use of enzymes in large-scale industrial applications, high-level expression of enzymes in recombinant hosts is usually a prerequisite. In the present work, two thermostable recombinant xylanases from the two thermophilic organisms (*N. flexuosa *and *T. aurantiacus*), which were cloned and expressed in *T. reesei*, were purified and characterized. The hydrolytic properties of the two xylanases on xylans and lignocellulosic substrates were also investigated. The main aim of the present study was to compare the biochemical and hydrolytic properties of two thermostable recombinant GH11 and GH10 xylanases, both lacking the CBM, with respect to two potential application areas: for the production of XOSs and for the hydrolysis of lignocellulosic substrates. Isolated xylan substrates and hydrothermally pretreated wheat straw, a potential substrate for generating platform sugars, were used to compare the enzymes.

## Methods

### Materials

Birchwood glucuronoxylan was purchased from Sigma Chemical Co. (St. Louis, MO, USA). Oat spelt arabinoxylan was obtained from Serva (Heidelberg, Germany), and hydrothermally pretreated wheat straw was a kind gift of Inbicon (Fredericia, Denmark). The dry matter (DM) content of this wheat straw after being washed was 35.8%, and the contents of glucan and xylan were 58.9% and 3.2%, respectively, as determined by high-pressure liquid chromatography (HPLC) using the analytical CarboPac PA-1 column (Dionex Corp., Sunnyvale, CA, USA. D-xylose, D-arabinose, D-glucose (Merck, Darmstadt, Germany), 1,4-β-D-xylobiose, 1,4-β-D-xylotriose and 1,4-β-D-xylotetraose (Megazyme, Bray, Ireland) were used as carbohydrate standards. Arabinoxylobiose (AX) [18], aldotetrauronic acid (U^4m2^XX) and aldopentauronic acid (XU^4m2^XX) (S. Koutaniemi, unpublished data) were prepared by enzymatic endoxylanase hydrolysis of rye arabinoxylan and birchwood glucuronoxylan, respectively, followed by chromatographic purification. The nomenclature for oligosaccharides derived from xylan was derived from the naming system described by Fauré *et al*. [19]. All other chemicals used were of analytical grade and purchased from Sigma or Merck.

### Purification of *N. flexuosa *and *T. aurantiacus *xylanases

The two recombinant xylanases from *N. flexuosa *and *T. aurantiacus *were kindly provided by Roal Oy (Rajamäki, Finland). The enzymes were produced in a genetically modified *T. reesei *strain using a method whereby the genes *cbh1, cbh2, egl1 and egl2 *encoding for Cel7A, Cel6A, Cel7B and Cel5A, respectively, had been deleted according to a method described elsewhere [16,17,20]. To remove the less thermostable enzymes produced by the host strain *T. reesei*, the two xylanase preparations were adjusted to pH 6.0 and were treated at 60°C for 2 hours. The supernatants were further purified by gel permeation chromatography. The column with Sephacryl S-200 (1.6 × 60 cm; GE Healthcare Life Sciences, Uppsala, Sweden) was preequilibrated and run in 50 mM sodium citrate buffer (pH 5.0) with the addition of 100 mM NaCl at a flow rate of 0.5 ml/minute. Fractions of 2 ml were collected and analyzed for xylanase activity.

### Enzyme analysis

Xylanase activity was assayed using 1% (wt/vol) birchwood glucuronoxylan (Roth 7500, Karlsruhe, Germany) as a substrate in 50 mM sodium citrate buffer according to the method of Bailey *et al*. [21]. The assay was performed at pH 5.0 and 50°C for 5 minutes. The amount of reducing sugars liberated was determined using the dinitrosalicylic acid (DNS) method with xylose used as standard [22]. One nanokatal (1 nkat) of the enzyme activity was defined as the amount of enzyme that catalyzes the release of 1 nM reducing sugar per second. Activities of β-xylosidase and β-glucosidase were assayed using 5 mM *p*-nitrophenyl-β-D-xylopyranoside (N2132; Sigma Chemical Co.) and 1 mM *p*-nitrophenyl-β-D-glucopyranoside (N7006; Sigma) as substrates, respectively [23,24]. Endoglucanase activity was determined using hydroxyl ethyl cellulose (Sigma-Aldrich, St. Louis, MO, USA) as substrate [25]. The filter paper activity (FPA) was determined using Whatman 1 filter paper as substrate [25]. All activities presented are average values of three separate determinations.

Protein was quantified by the Lowry method using bovine serum albumin (BSA) (Sigma Chemical Co.) as standard [26]. Proteins in the column eluents were monitored by measuring the absorbance at 280 nm. The molecular masses of the purified xylanases were determined by matrix-assisted laser desorption/ionization time-of-flight mass spectrometry (MALDI-TOF-MS) (Bruker Daltonik, Bremen, Germany) at the Institute of Biotechnology (Helsinki, Finland). Sodium dodecyl sulfate polyacrylamide gel electrophoresis (SDS-PAGE) was performed on 12% polyacrylamide gel using the method of Laemmli [27]. A prestained protein ladder (Invitrogen, Carlsbad, CA, USA) was used as a molecular weight standard. After electrophoresis, the gel was stained with Coomassie brilliant blue G-250 (Bio-Rad, Hercules, CA, USA).

N-terminal amino acid sequences of the enzymes were performed by Edman degradation at the Institute of Biotechnology (Helsinki, Finland). Apparent kinetic parameters were determined by incubating purified xylanases with different concentrations of birchwood glucuronoxylan in 50 mM sodium citrate buffer at pH 5.0 and 50°C. The Michaelis maximum velocity (*V*_max_) and constant (*K*_m_) values were determined by using the double-reciprocal plot method of Lineweaver-Burk.

### Characterization of xylanases

The optimal pH for xylanase activity was determined by using McIlvaine buffer between pH 3 and pH 8 (50°C), and the optimal temperature for xylanase activity was determined to be between 30°C and 85°C (pH 5.0). The thermal stability of xylanases was determined after incubating the Nf Xyn11A (20.1 mg/mL) and Ta Xyn10A (20.1 mg/mL) samples without substrate in 50 mM sodium acetate buffer (pH 5.0) at 70°C and 80°C. The samples were removed after incubation for varying times (30 minutes and 1, 2, 3 and 4 hours) and immediately cooled on ice. The residual xylanase activity was determined using the standard assay.

### Hydrolytic properties on polysaccharides

The hydrolysis of birchwood glucuronoxylan and insoluble oat spelt arabinoxylan (9 mg/mL) was carried out in test tubes with a working volume of 1 mL. Insoluble oat spelt arabinoxylan was prepared by using a modified method of Ryan *et al*. [28]. Oat spelt arabinoxylan (4 g) was suspended in 400 mL of distilled water and stirred overnight at room temperature. The insoluble fraction was recovered by centrifugation for 20 minutes at 10,000 rpm and 4°C. The insoluble fraction was washed several times with Milli-Q water (Milli-Q Plus; Millipore, Billerica, MA, USA). After that, the sediment was lyophilized and used for hydrolysis. The enzyme dosage was 0.22 mg protein/g substrate. The hydrolysis of xylan substrates was carried out in 50 mM sodium citrate buffer (pH 5.0) at 55°C. Aliquots were removed periodically at different time intervals and boiled for 10 minutes to stop the enzymatic hydrolysis. Reducing sugars in supernatants were determined by using the DNS method [22]. Xylose standard was used for calibration. Two replicates were carried out, and average values are presented.

To investigate the end products, birchwood glucuronoxylan and oat spelt arabinoxylan (5 g/L) were incubated with Nf Xyn11A (1.71 mg protein/g substrate, equal to 10,000 nkat/g substrate) and Ta Xyn10A (0.90 mg protein/g substrate, equal to 10,000 nkat/g substrate) in 50 mM sodium citrate buffer, pH 5.0, at 50°C for 48 hours, respectively. Hydrolysis was terminated by boiling the samples in a water bath for 10 minutes, after which the samples were analyzed by performing thin-layer chromatography (TLC) and high-performance anion exchange chromatography coupled with pulsed amperometric detection (HPAEC-PAD).

The hydrolysis of the hydrothermally pretreated, washed wheat straw was carried out by Nf Xyn11A and Ta Xyn10A in test tubes with a working volume of 2 mL in 50 mM sodium citrate buffer (pH 5.0) containing 0.02% NaN_3 _at 55°C. The DM content of substrate was 2%, and xylanases were dosed at 1 mg protein/g substrate. Samples were withdrawn at 4, 8 and 24 hours and boiled for 10 minutes to stop the enzymatic hydrolysis. After cooling, the samples were centrifuged and the supernatants were analyzed for reducing sugars using the DNS method with xylose as standard [22].

### Carbohydrate analysis

The hydrolysis products containing xylose and XOS were analyzed using TLC. TLC was carried out using Silica Gel 60 plates (Merck). A mixture of ethyl acetate, acetic acid, isopropanol, formic acid and water (25:10:5:1:15 vol/vol) was used as the running solution for analyzing hydrolysates of birchwood glucuronoxylan. A mixture of 1-butanol, ethanol and water (3:2:2 vol/vol) was used as the running solution for analyzing the hydrolysates of oat spelt arabinoxylan. The carbohydrates were detected by spraying with 2% (wt/vol) orsinol dissolved in a solution of ethanol, H_2_SO_4 _and H_2_O (80:10:10%), after which the plates were heated at 100°C for 10 minutes.

Oligosaccharides in the hydrolysates from birchwood glucuronoxylan and oat spelt arabinoxylan were analyzed using HPAEC-PAD [29]. The HPAEC-PAD system was equipped with an SSI pulse equalizer (model LP 21; Scientific Systems, Inc., State College, PA, USA), two Waters 515 HPLC pumps, a PC Waters pump control module and a cooling Waters 717 autosampler using Millenium software (Waters Corp., Milford, MA, USA) for instrument control and data handling. The analytical CarboPac PA-100 column (250 × 4 mm) and the guard column PA-100 (25 × 3 mm) (Dionex Corp.) were maintained at 30°C. The eluents for gradient analysis were 100 mM NaOH and 100 mM NaOH/1 M NaOAc at a flow rate of 1 mL/minute as described by Rantanen *et al*. [18]. D-xylose, linear XOS (X_2_-X_4_) and substituted XOS (AX, U^4m2^XX and XU^4m2^XX) were used as external standards.

## Results and discussion

### Purification of xylanases

**Figure 1:**
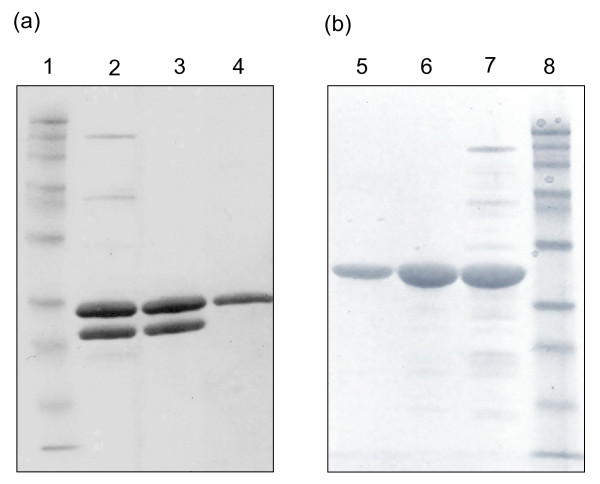
**Sodium dodecyl sulfate polyacrylamide gel electrophoresis (SDS-PAGE) of Nf Xyn11A and Ta Xyn10A**. **(a) **Lane 1: Molecular mass markers. Lane 2: *N. flexuosa *xylanase preparation. Lane 3: *N. flexuosa *xylanase preparation after heat treatment. Lane 4: Purified Nf Xyn11A. **(b) **Lane 8: Molecular mass markers. Lane 7: *T. aurantiacus *xylanase preparation. Lane 6: *T. aurantiacus *xylanase preparation after heat treatment. Lane 5: Purified Ta Xyn10A. Sizes of molecular mass markers in (a) and (b) from top to bottom are 180, 115, 82, 64, 49, 37, 26, 19, 15 and 6 kDa.

The recombinant xylanases from *N. flexuosa *(Xyn11A) and *T. aurantiacus *(Xyn10A) expressed in *T. reesei *were heat-treated at 60°C and pH 6.0 for 2 hours. The enzymatic activities of the two preparations before and after heat treatment are shown in Table 1. There was no obvious decrease of xylanase activity in the two enzyme preparations after the heat treatment. After the heat treatment, however, the activities of β-xylosidase, β-glucosidase, endoglucanase and FPA of the *N. flexuosa *xylanase preparation decreased significantly. These activities were due to the enzymes produced by the host strain *T. reesei *and were, as expected, not stable at 60°C. The protein concentration in the xylanase preparation of *N. flexuosa *after heat treatment was almost the same as before heat treatment, indicating the high expression level of xylanase in the preparation (Figure 1). Similar phenomena regarding the enzymatic activities were observed in the xylanase preparation of *T. aurantiacus*. The protein concentration of the preparation decreased slightly from 24.1 to 20.1 mg/mL after the heat treatment, which indicated that some less thermostable proteins were removed after the heat treatment (Figure 1). The measured β-xylosidase activity was obviously due to the reported activity of the family 10 enzyme toward the substrate of the β-xylosidase [30,31]. The only remaining activities originating from the *T. reesei *strain were those of endoglucanases.

**Table 1:** Enzymatic activities of recombinant xylanase preparation from *N. flexuosa *(Nf), xylanase preparation from *T. aurantiacus *(Ta) and the corresponding two preparations after heat treatment for 2 hours at pH 6.0 and 60°C (Nf-H, Ta-H)

Preparation	Protein (mg/mL)	Xylanase (nkat/mL)	β-xylosidase (nkat/mL)	β-glucosidase (nkat/mL)	Endoglucanase (nkat/mL)	FPA (FPU/mL)	Family of GH
Nf	20.1	112999	6.0	19.5	55.4	0.2	11
Nf-H	20.1	117263	0	0.9	30.5	0	
Ta	24.1	230368	49. 8	72.3	140.5	0.2	10
Ta-H	20.1	223464	43.3	2.9	37.8	0	

Gel permeation chromatography was used for further purification. Two peaks with xylanase activities were obtained when the xylanase preparation from *N. flexuosa *after heat treatment was fractionated by Sephacryl S-200. The peak with higher molecular weight was collected. A single peak with xylanase activity was obtained and collected from the xylanase preparations from *T. aurantiacus *after heat treatment by the same purification system. Both purified xylanases presented electrophoretic homogeneity by SDS-PAGE (Figure 1), which permitted physicochemical characterization of these enzymes.

### Molecular weight and N-terminal amino acid sequence

Table 2 summarizes some physicochemical and kinetic characteristics of the purified xylanases from *N. flexuosa *(Nf Xyn11A) and *T. aurantiacus *(Ta Xyn10A). The molecular weights of Nf Xyn11A and Ta Xyn10A were estimated to be approximately 25 kDa and 32 kDa on the basis of SDS-PAGE (Figure 1). The molecular weights of Nf Xyn11A and Ta Xyn10A were determined to be 23,439 Da and 32,825 Da, respectively, by MALDI-TOF-MS. The molecular weight of the Nf Xyn11A corresponded to the shorter form of the enzyme containing the catalytic domain associated with the peptide linker but lacking the C-terminal CBM, and the molecular weight of the Ta Xyn10A corresponded to the native enzyme [16,17,32]. The Ta Xyn10A did not have a CBM either, and therefore these two enzymes were comparable on solid substrates. The N-terminal amino acid sequence of Nf Xyn11A was determined by Edman degradation to be DTTITQ, which was in accordance with the construct [33]. The N-terminal amino acid sequence of Ta Xyn10A was not available by Edman degradation, because it was blocked. Such block may be caused by the acetyl group, which has previously been detected in a xylanase from the culture media of *T. aurantiacus *[34].

**Table 2:** Biochemical properties of recombinant Nf Xyn11A and Ta Xyn10A xylanases^a^

Enzymes	Specific activity (nkat/mg)	Mw (MS) (kDa)	*K*_m_ (mg/mL)	*V*_max_ (nkat/mg)	*k*_cat _(s^-1^)	*k*_cat_/*K*_m_ (mg^-1 ^s^-1 ^ml)
Nf Xyn11A	3,064	23.4	6.0	5,862	136.9	22.8
Ta Xyn10A	13,691	32.8	1.0	7,236	236.9	236.9

^a^Kinetic values were analyzed using birchwood glucuronoxylan as substrate.

### Kinetic properties

The apparent kinetic parameters of Nf Xyn11A and Ta Xyn10A enzymes were determined at 50°C and pH 5.0 by using birchwood glucuronoxylan as substrate. For Nf Xyn11A, the values of *V*_max _and *K*_m _determined from Lineweaver-Burk plots were 5,862 nkat/mg and 6.0 mg/mL (Table 2). The *K*_m _of Nf Xyn11A was similar to the short proteins of r23.8 kDa and r22.0 kDa produced in *T. reesei *[16]. The specific activity of Nf Xyn11A was 3,064 nkat/mg, which was lower than the r23.8 kDa reported by Leskinen *et al*. [16]. The specific activity of Ta Xyn10A was 13,691 nkat/mg, which was higher than the activity of xylanase from *T. aurantiacus *Miehe, IMI 216529 and *T. aurantiacus *ALKO4242 [14,17]. The *K*_m _of the Ta Xyn10A was 1.0 mg/mL, which was close to that of the GH10 xylanase from *Streptomyces sp*. QG-11-3 [35]. The lower *K*_m _of Ta Xyn10A indicated that Ta Xyn10A had higher affinity than Nf Xyn11A for birchwood glucuronoxylan. The Ta Xyn10A had also a higher turnover number *k*_cat _and catalytic efficiency *k*_cat_/*K*_m_, indicating high catalytic activity.

### Effect of pH and temperature on activity and thermostability

**Figure 2:**
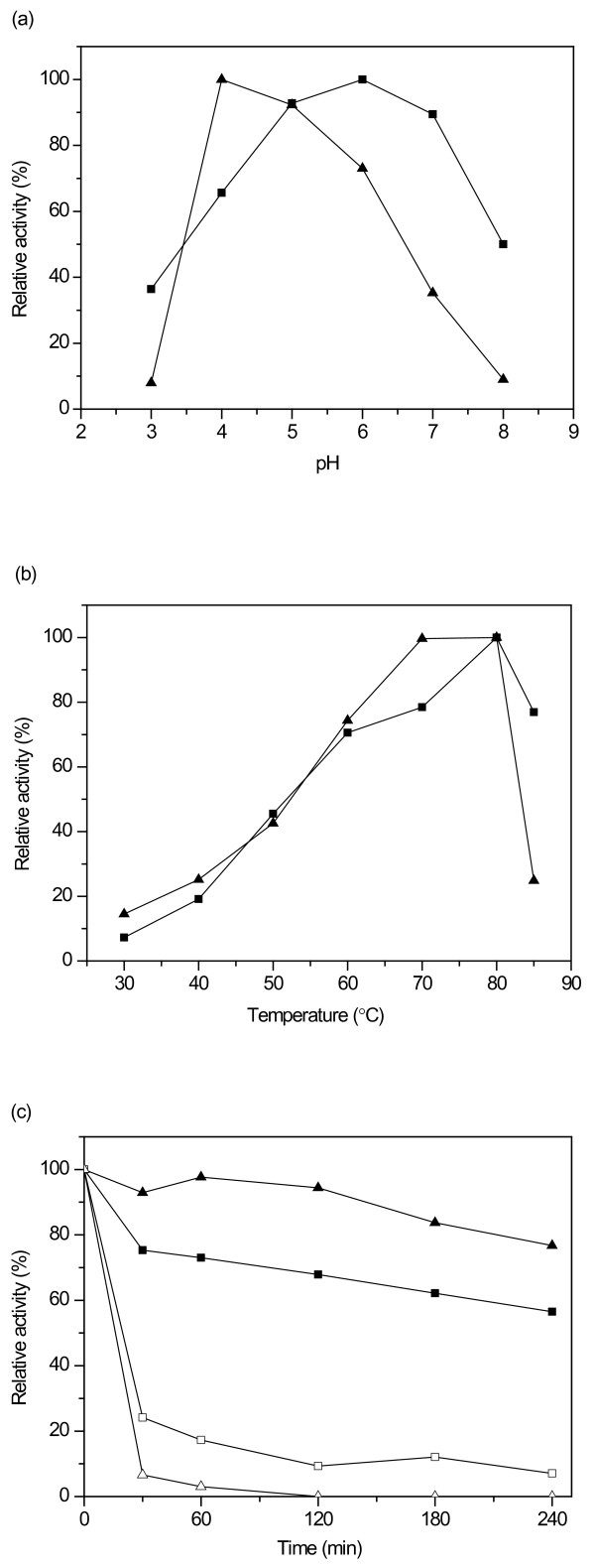
**Effect of (a) pH (at 50°C) and (b) temperature (at pH 5.0) on xylanase activities of Nf Xyn11A (filled square) and Ta Xyn10A (filled triangle) and (c) their thermostabilities**. Xylanase activity determinations were carried out by 5-minute incubation at different pH values and temperatures. The values of relative activities are the means of three replicates. Residual activity was measured after incubating the enzyme at 70°C (closed symbols) and 80°C (open symbols) in the absence of substrate.

The xylanase activities of Nf Xyn11A and Ta Xyn10A enzymes at various pH values were measured by using birchwood xylan as substrate without addition of stabilizing BSA. The Nf Xyn11A showed maximal activity at pH 6 (50°C) and maintained good activity from pH 5 to pH 7 (Figure 2a). All measurements of activities had a relative standard deviation lower than 5%. At pH 3 the activity was 36%of the maximal activity, and at pH 8 the activity was approximately 50% of the maximal activity. Ta Xyn10A showed maximal activity at pH 4 and pH 5. At pH 3 and pH 8, it lost approximately 92% and 91% of the maximal activity, respectively. The highest xylanase activity of Nf Xyn11A was detected at 80°C. The enzyme still maintained over 75% of the maximal activity at 85°C (Figure 2b). Ta Xyn10A exhibited its optimal activity at 70°C to 80°C, but the activity decreased rapidly at 85°C.

The thermal stabilities of the two xylanases were determined at 70°C and 80°C in the absence of substrate (Figure 2c). Ta Xyn10A had good stability at 70°C and retained approximately 77% of the activity after incubation for 4 hours. At 80°C, the xylanase activity of Ta Xyn10A decreased rapidly, and all activity was lost after 2 hours of incubation. Nf Xyn11A had slightly lower thermal stability than Ta Xyn10A at 70°C but, somewhat surprisingly, higher thermal stability than that of Ta Xyn10A at 80°C.

### Mode action of the two xylanases on xylans

#### Hydrolysis of isolated xylans

**Figure 3:**
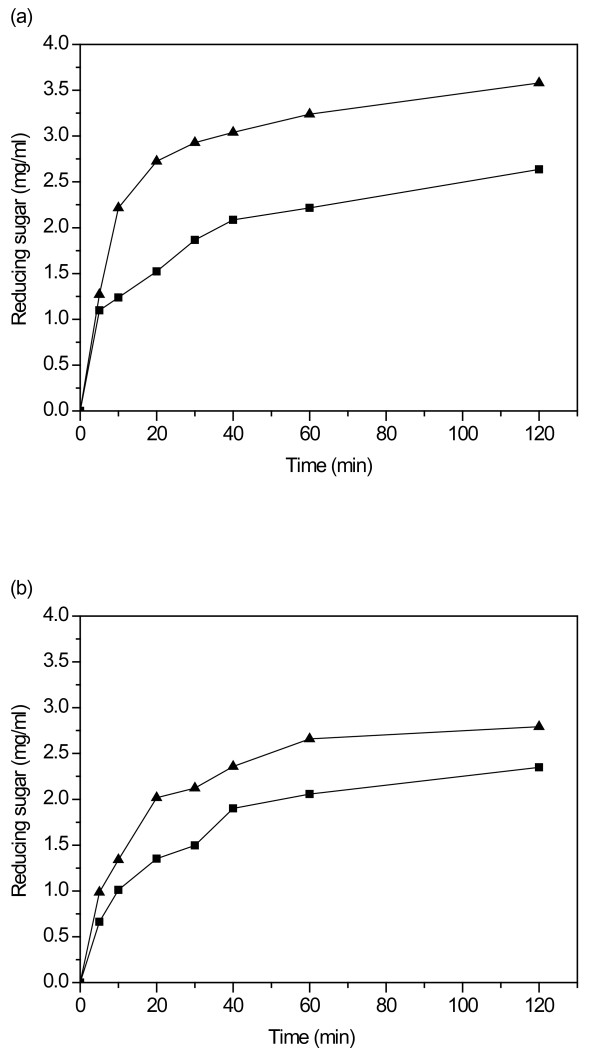
**Hydrolysis on (a) birchwood glucuronoxylan and (b) insoluble oat spelt arabinoxylan by Nf Xyn11A (filled squares) and Ta Xyn10A (filled triangles)**. Hydrolysis was conducted in 50 mM sodium citrate buffer with a dosage of 0.22 mg protein/g substrate at pH 5.0 and at 55°C.

Two isolated xylan substrates, birchwood glucuronoxylan and insoluble oat spelt arabinoxylan, were chosen as well-characterized reference substrates to compare the hydrolytic properties of the two enzymes. Birchwood glucuronoxylan is substituted by the 4-*O*-methylglucuronic acid and acetyl groups. In comparison, the insoluble oat spelt arabinoxylan is mainly decorated with arabinofuranose side groups. The hydrolysis of birchwood glucuronoxylan and insoluble oat spelt arabinoxylan was followed first by the release of reducing sugars (Figure 3). The enzymes were dosed at equal amounts on the protein basis (0.22 mg protein/g substrate, 674 nkat/g substrate of Nf Xyn11A and 3,012 nkat/g substrate of Ta Xyn10A). Clear differences in the amounts of reducing sugars released by Nf Xyn11A and Ta Xyn10A from soluble and insoluble xylans at 55°C and pH 5.0 were detected. The Ta Xyn10A released 3.6 mg/mL reducing sugars from birchwood glucuronoxylan after 2 hours of hydrolysis, which was higher than that released by Nf Xyn11A (2.6 mg/mL) (Figure 3a). Ta Xyn10A released significantly more reducing sugars than Nf Xyn11A, also from insoluble oat spelt arabinoxylan, after 2 hours of hydrolysis (Figure 3b). The results indicate that Ta Xyn10A was more efficient than Nf Xyn11A in the hydrolysis of both birchwood glucuronoxylan and insoluble oat spelt arabinoxylan. The detected difference in hydrolysis efficiency on xylans by these two enzymes may be partly due to the fact that Nf Xyn11A was more severely hampered by the presence of substituents in xylans than Ta Xyn10A, in accordance with the findings reported in previous studies [4,31,36-38]. The enzymes were compared on the protein basis, and the results also reflected the higher specific activity of the Ta Xyn10A enzyme. As expected, both enzymes produced a higher degree of hydrolysis on the birchwood glucuronoxylan. Similar results have been reported for other xylanases. The hydrolysis of soluble and insoluble beechwood xylan (or wheat straw xylan) was compared with an endoxylanase from *Penicillium capsulatum*, and a higher degree of hydrolysis was always obtained on soluble substrates than on insoluble substrates [28]. The hydrolysis of soluble and insoluble oat spelt arabinoxylan was also investigated by using two xylanases from *Streptomyces olivaceoviridis *E-86, and a higher degree of hydrolysis was obtained from the soluble substrate [39].

**Figure 4:**
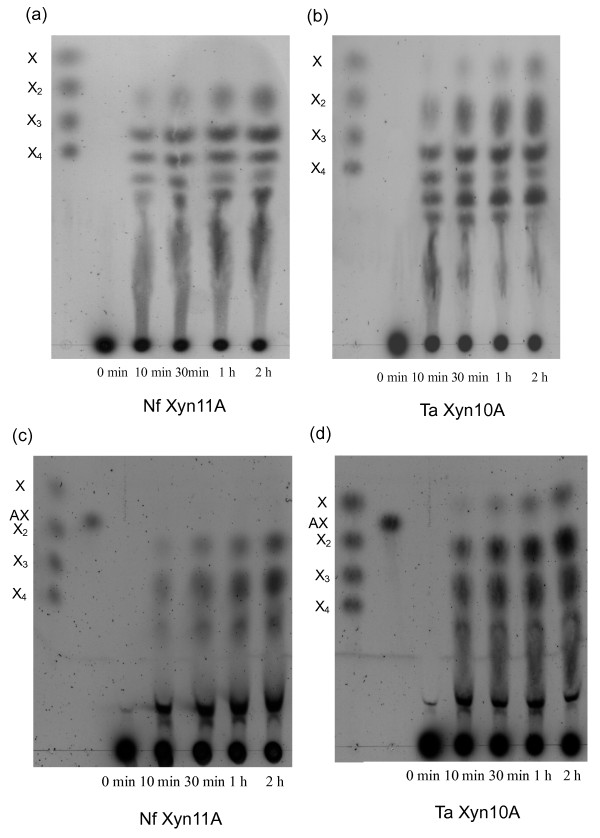
**Thin-layer chromatography (TLC) analysis of hydrolysis products released from (a and b) birchwood glucuronoxylan and (c and d) insoluble oat spelt arabinoxylan**. Hydrolysis was conducted by Nf Xyn11A and Ta Xyn10A enzymes after different incubation times. Xylose to xylotetraose and arabinoxylobiose were used as standards. X, xylose; X_2_, xylobiose; X_3_, xylotriose; X_4_, xylotetraose; AX, arabinoxylobiose. The buffer salts retard the sample elution compared to the standards.

Formation of hydrolysis products during 2 hours of incubation was followed by TLC (Figure 4). The initial hydrolysis products released from both birchwood glucuronoxylan and oat spelt arabinoxylan by Nf Xyn11A and Ta Xyn10A contained oligomers from xylobiose to xylopentaose and other, longer, unidentified XOSs, indicating typical endoxylanase action. The two xylanases differed in the production of xylose, as a very low amount of xylose was formed during the first hour of the hydrolysis by Nf Xyn11A (Figures 4a and 4c), whereas xylose was clearly detected in the Ta Xyn10A hydrolysates (Figures 4b and 4d). This indicated that Nf Xyn11A could be a better potential enzyme than Ta Xyn10A for the production of XOS.

#### End products of xylan hydrolysis

**Figure 5:**
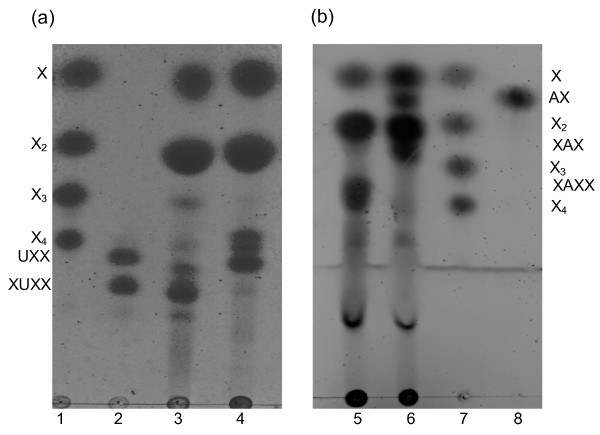
**TLC analysis of the end point hydrolysis products from (a) birchwood glucuronoxylan and (b) insoluble oat spelt arabinoxylan by Nf Xyn11A and Ta Xyn10A**. (a) Lane 1: Standards X−X_4_. Lane 2: Standards MeGlcA^3^Xyl_3 _(UXX) and MeGlcA^3 ^Xyl_4 _(XUXX). Lane 3: Hydrolysis products by Nf Xyn11A. Lane 4: Hydrolysis products by Ta Xyn10A. (b) Lane 5: Hydrolysis products by Nf Xyn11A. Lane 6: Hydrolysis products by Ta Xyn10A. Lane 7: Standards X-X_4_. Lane 8: Standard arabinoxylobiose (AX). To make the position of hydrolysis products more clear, the positions of XAX and XAXX are also indicated.

**Figure 6:**
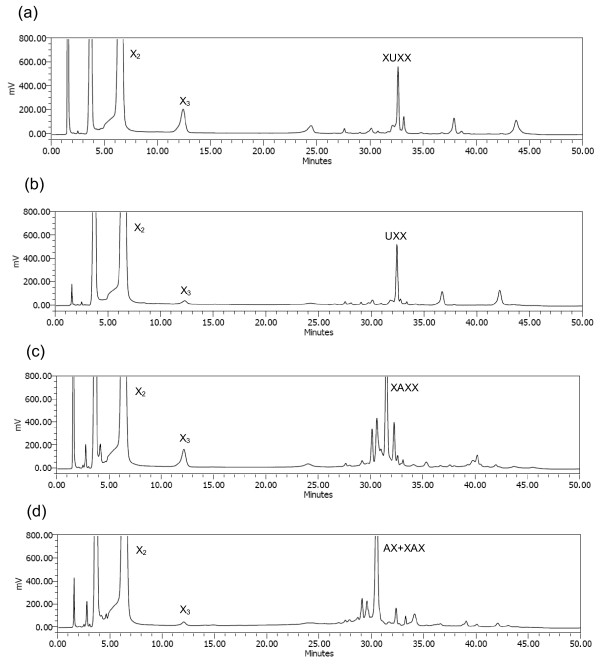
**High-performance anion exchange chromatography coupled with pulsed amperometric detection (HPAEC-PAD) of hydrolysates on (a and b) birchwood glucuronoxylan and (c and d) insoluble oat spelt arabinoxylan by (a and c) Nf Xyn11A and (b and d) Ta Xyn10A enzymes**. X_2_, xylobiose; X_3_, xylotriose; XUXX, MeGlcA^3^Xyl_4_; UXX, MeGlcA^3^Xyl_3_; XAXX, arabinoxylotetraose; XAX, arabinoxylotriose, AX, arabinoxylobiose. The peaks at about 4 minutes correspond to xylose. The peak of XAXX was tentatively identified from the order to p*K*_a _values of different arabinoxylobiose xylooligosaccharide (AXOS) and the shortest AXOS from GH11 xylanases.

The hydrolysis products of the birchwood glucuronoxylan and oat spelt arabinoxylan with Nf Xyn11A and Ta Xyn10A enzymes (10,000 nkat/g) after prolonged hydrolysis of 48 hours at 50°C were analyzed by TLC (Figure 5) and HPAEC-PAD (Figure 6). Both xylanases produced xylose and xylobiose as the major end products from birchwood glucuronoxylan and oat spelt arabinoxylan, but more xylotriose was detected in Nf Xyn11A than in Ta Xyn10A hydrolysate (Figures 5 and 6). This action was in accordance with those of *T. fusca *xylanases Xyl11A and Xyl10B [40]. Xylose was clearly detected among the end products by Nf Xyn11A, although xylose content was still higher in Ta Xyn10A hydrolysates (Figure 5). The hydrolysis products released from birchwood xylan and oat spelt xylan by the recombinant Xyl10B and Xyl11A xylanases from *Thermobifida fusca *have also been investigated previously [40]. The results showed that the GH10 (Xyl10B) xylanase produced more xylose than the GH11 (Xyl11A) xylanase on both xylan substrates, which is in accordance with the present results on Nf Xyn11A and Ta Xyn10A xylanases.

Nf Xyn11A released aldopentauronic acid (β-D-Xyl*p*-(1→4) [4-*O*-methyl-α-D-Glc*p*A-(1→2)]-β-D-Xyl*p*-(1→4)-β-D-Xyl*p *-(1→4)-D-Xyl, XU^4m2^XX) as the shortest acidic fragment from birchwood glucuronoxylan (Figures 5a and 6a). The main acidic end product released by Ta Xyn10A was aldotetrauronic acid (4-*O*-methyl-α-D-Glc*p*A-(1→2)-β-D-Xyl*p*-(1→4)-β-D-Xyl*p*-(1→4)-D-Xyl, U^4m2^XX) (Figures 5a and 6b). The other two minor acidic oligosaccharides, eluting later in the HPAEC-PAD (between 35 and 45 minutes) corresponded to XOSs carrying 4-*O*-methyliduronic acid and hexenuronic acid, respectively, which were formed from the 4-*O*-methylglucuronic acid during the xylan isolation [41,42]. Thus both enzymes acted on glucuronoxylan as typical GH11 and GH10 endoxylanases [4,43].

The Nf Xyn11A enzyme released arabinoxylotetraose (β-D-Xyl*p*-(1→4)[α-L-Ara*f*-(1→3)]-β-D-Xyl*p*-(1→4)-D-Xyl*p*-(1→4)-D-Xyl, XA^3^XX) as the shortest arabinoxylan oligosaccharides (AXOS) from oat spelt arabinoxylan, whereas arabinoxylobiose (α-L-Ara*f*-(1→3)-β-D-Xyl*p*-(1→ 4)-D-Xyl*p*, A^3^X) was the shortest AXOS liberated by Ta Xyn10A (Figures 5 and 6). For more precise identification of the shortest AXOS formed, TLC analysis would need to be performed, as some of the AXOSs, such as A^3^X and XA^3^X, coelute in the HPAEC-PAD [18]. Thus, in addition to A^3^X, a longer XA^3^X was also clearly identified as a second AXOS end product by Ta Xyn10A (Figure 5b). The retention time in HPAEC-PAD is inversely correlated with p*K*_a _value and increases with increasing molecular mass of the carbohydrate. The peak of XA^3^XX shown in Figure 6c was tentatively identified on the basis of the longer retention time compared with A^3^X and XA^3^X, the elution order of different AXOSs and being the shortest AXOS from GH11 xylanases. It was previously reported that XA^3^XX was the shortest AXOS released from arabinoxylan by GH11 xylanase from *Thermomyces lanuginosus *[44] and that A^3^X was the shortest AXOS from arabinoxylan by GH10 xylanase from *Aspergillus aculeatus *[18]. These results are in accordance with present observations on the shortest AXOS formed from arabinoxylan by the Nf Xyn11A and Ta Xyn10A xylanases, respectively (Figures 5b, 6c and 6d). The Ta Xyn10A always released shorter acid XOSs and AXOSs from substituted xylans. Our results also confirmed that the endoxylanases from GH10 cleave the glycosidic linkages in the xylan main chain closer to the substituent, resulting in shorter products [4,31,37].

### Hydrolytic properties on wheat straw

**Figure 7:**
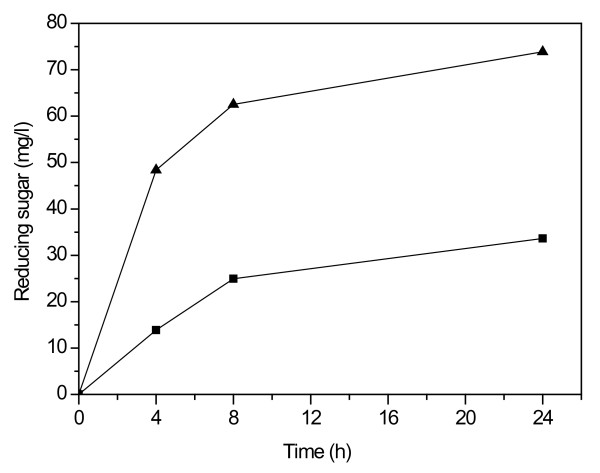
**Reducing sugars released from hydrothermally pretreated, washed wheat straw**. Hydrolysis was conducted by Nf Xyn11A and Ta Xyn10A, 1 mg protein/g substrate, in 50 mM sodium citrate buffer after 4, 8 and 24 hours of hydrolysis, substrate consistency 2% and pH 5.0 at 55°C.

Finally, the properties of the two xylanases were compared (enzyme dosage 1 mg protein/g DM) on the hydrolysis of hydrothermally pretreated, washed wheat straw on the basis of formation of reducing sugars (Figure 7). Ta Xyn10A produced more reducing sugars also from the wheat straw substrate than Nf Xyn11A, indicating the higher hydrolysis efficiency of Ta Xyn10A also on xylan-containing lignocellulosic substrates (Figure 3). Previously, the GH10 xylanase from *T. fusca *has also been shown to have higher xylanase activity than the GH11 xylanase on the lignocellulosic substrate corn stover [40]. However, it has also been reported that the GH11 xylanase was more efficient than the GH10 xylanase in the hydrolysis of wheat bran [45]. The Ta Xyn10A alone was able to release 73.9 mg/L reducing sugars from the lignocellulosic substrate within 24 hours using a dosage of 1 mg protein/g DM, as compared with release 33.6 mg/L reducing sugars by Nf Xyn11A. Generally, the hydrolysis products of lignocellulosic substrates by xylanases contained xylose and XOSs of DP 2 to 4. The detailed structure of the residual xylan in pretreated wheat straw has not been characterized in detail. The pretreatment conditions affect profoundly the amount and structure of xylan, including the degree of substitution. On the basis of the present data, the hydrolytic capabilities of the two enzymes on the pretreated wheat straw have been shown to resemble those on the isolated xylan substrates. The present results clearly indicate that the GH10 xylanase from *T. aurantiacus *has a better ability than the *N. flexuosa *xylanase to hydrolyse xylan also in lignocellulosic biomass.

## Conclusions

The two purified recombinant xylanases were shown to have good thermal stability at 70°C. As compared on the protein basis, the xylanase from *T. aurantiacus *was more efficient than the *N. flexuosa *xylanase in hydrolysis of isolated xylans and lignocellulosic biomass. The mode of action of the xylanases from *T. aurantiacus *and *N. flexuosa *on xylan was typical of enzymes belonging to the GH10 and GH11 families, respectively. Because of its high catalytic activity and good thermostability, the *T. aurantiacus *xylanase shows great potential for applications aimed at total hydrolysis of lignocellulosic materials for platform sugars, whereas the *N. flexuosa *xylanase has better potential as an enzyme for the production of XOSs.

## Abbreviations

AX: arabinoxylobiose; AXOS: arabinoxylan oligosaccharide; CBM: cellulose-binding module; DM: dry matter; GH: glycosyl hydrolase family; HPAEC-PAD: high-performance anion exchange chromatography coupled with pulsed amperometric detection; TLC: thin-layer chromatography; XAX: arabinoxylotriose; XAXX: arabinoxylotetraose; XOS: xylooligosaccharide; UXX: aldotetrauronic acid; XUXX: aldopentauronic acid.

## Competing interests

The authors declare that they have no competing interests.

## Authors' contributions

JZ designed and coordinated the experimental work, analyzed the results and drafted the manuscript. MS, TP and MTa reviewed the paper. MTe designed the work, analyzed the results and reviewed the paper. LV conceived and coordinated the overall study and helped to analyze the results and finalize the paper. All authors read and approved the final manuscript.
